# Discriminating electrocardiographic responses to His-bundle pacing using machine learning

**DOI:** 10.1016/j.cvdhj.2020.07.001

**Published:** 2020-08-26

**Authors:** Ahran D. Arnold, James P. Howard, Aiswarya Gopi, Cheng Pou Chan, Nadine Ali, Daniel Keene, Matthew J. Shun-Shin, Yousif Ahmad, Ian J. Wright, Fu Siong Ng, Nick W.F. Linton, Prapa Kanagaratnam, Nicholas S. Peters, Daniel Rueckert, Darrel P. Francis, Zachary I. Whinnett

**Affiliations:** National Heart and Lung Institute, Imperial College London, Hammersmith Hospital, London, United Kingdom

**Keywords:** Artificial intelligence, Conduction system pacing, Electrocardiography, His-bundle pacing, Machine learning, Neural networks, Pacemakers

## Abstract

**Background:**

His-bundle pacing (HBP) has emerged as an alternative to conventional ventricular pacing because of its ability to deliver physiological ventricular activation. Pacing at the His bundle produces different electrocardiographic (ECG) responses: selective His-bundle pacing (S-HBP), non-selective His bundle pacing (NS-HBP), and myocardium-only capture (MOC). These 3 capture types must be distinguished from each other, which can be challenging and time-consuming even for experts.

**Objective:**

The purpose of this study was to use artificial intelligence (AI) in the form of supervised machine learning using a convolutional neural network (CNN) to automate HBP ECG interpretation.

**Methods:**

We identified patients who had undergone HBP and extracted raw 12-lead ECG data during S-HBP, NS-HBP, and MOC. A CNN was trained, using 3-fold cross-validation, on 75% of the segmented QRS complexes labeled with their capture type. The remaining 25% was kept aside as a testing dataset.

**Results:**

The CNN was trained with 1297 QRS complexes from 59 patients. Cohen kappa for the neural network’s performance on the 17-patient testing set was 0.59 (95% confidence interval 0.30 to 0.88; *P* <.0001), with an overall accuracy of 75%. The CNN’s accuracy in the 17-patient testing set was 67% for S-HBP, 71% for NS-HBP, and 84% for MOC.

**Conclusion:**

We demonstrated proof of concept that a neural network can be trained to automate discrimination between HBP ECG responses. When a larger dataset is trained to higher accuracy, automated AI ECG analysis could facilitate HBP implantation and follow-up and prevent complications resulting from incorrect HBP ECG analysis.

## Graphical abstract

**Figure:**
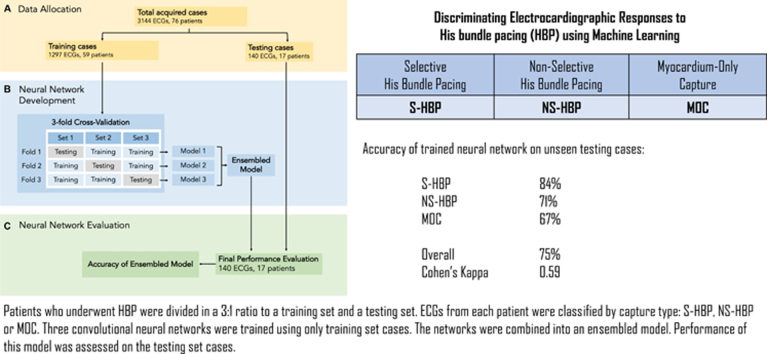

**Key Findings**

• The 3 main responses to His-bundle pacing are selective His-bundle pacing, non-selective His-bundle pacing, and myocardium-only capture. These responses must be distinguished from each other when performing His-bundle lead implantation and follow-up.
• His-bundle pacing electrocardiogram (ECG) response discrimination can be automated by training a neural network on labeled cases, a form of supervised machine learning.
• The neural network was best at differentiating between selective His-bundle pacing and myocardium-only capture and worst at differentiating between non-selective His-bundle pacing and myocardium-only capture, which is similar to what is expected from their distinguishing ECG features.
• Saliency mapping can reveal insights into how the neural network performs classification.

## Introduction

His-bundle pacing (HBP), in which the His-Purkinje cardiac conduction system is directly stimulated by a permanent pacing lead,^1^ has emerged as an alternative to conventional ventricular pacing, such as right ventricular (RV) apical pacing and biventricular pacing, in which only myocardium is stimulated. HBP can preserve physiological activation in patients with an intact conduction system and even can correct bundle branch block (BBB) to restore normal ventricular activation.^2^, ^3^, ^4^, ^5^, ^6^

The electrocardiogram (ECG) is a key tool for identifying successful pacing. Conventional ventricular pacing produces characteristic, easily discernible changes in the 12-lead ECG. RV apical pacing results in broad QRS complexes with left bundle branch block (LBBB) morphology.^7^ Ventricular pacing has been standard practice for decades and has resulted in a high global prevalence of experienced staff competent at interpreting paced ECGs recorded from conventional pacing locations.

**Figure 1:**
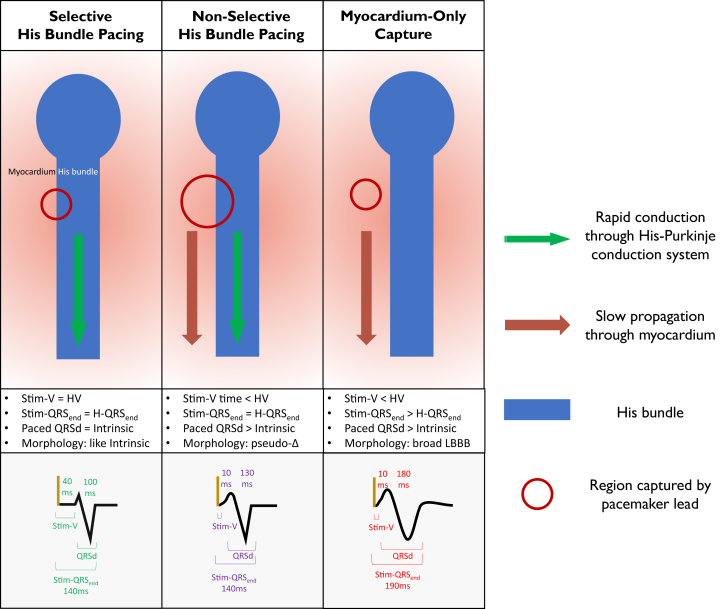
Electrocardiographic (ECG) responses to His-bundle pacing (HBP). Mechanisms and criteria for ECG responses to HBP. **Top row**: Variable tissue capture. **Middle row**: Key diagnostic features for narrow QRS HBP. **Bottom row**: Example measurements for narrow QRS HBP. H-QRS_end_ = time from His signal to QRS offset; HV = time from His signal to onset of QRS; LBBB = left bundle branch block; pseudo-Δ = pseudo–delta wave; QRSd = QRS duration; Stim-QRS_end_ = time from pacing artifact to QRS offset; Stim-V = time from pacing artifact to onset of QRS.

The emergence of HBP, however, has been accompanied by considerable challenges in ECG interpretation. Several ECG responses are potentially observed with HBP, and their differences can be subtle to the untrained eye (Figure 1 and Online Supplemental Appendix).

• *Selective HBP* (S-HBP) occurs when the His bundle is captured alone without any local myocardial capture.
• *Non-selective HBP* (NS-HBP) occurs when both the His bundle and local myocardium adjacent to the lead tip are captured.
• *Myocardium-only capture* (MOC) occurs when the His bundle is not captured and only the local myocardium is captured; this is also termed myocardial capture or septal capture.

Discriminating among these 3 ECG responses to HBP is more complex than ECG interpretation of conventional pacing, in which only the presence of ventricular capture needs to be identified. This complexity is compounded by a lack of expertise. Although its global uptake has been rapid, as a relatively recent development HBP implantation and follow-up are performed at only a small number of centers. Discriminating among the 3 responses has crucial clinical importance, as misdiagnosis can have adverse consequences. During HBP lead implantation, operators must rapidly determine whether the achieved lead position is satisfactory (capturing at a reasonable threshold) or the lead must be repositioned. At follow-up, appropriate pacing output and configuration must be selected to achieve the desired capture type.

Machine learning is a form of artificial intelligence (AI) that allows automation of tasks that otherwise would require human expertise, including ECG classification.^8^, ^9^, ^10^, ^11^, ^12^ Automated analysis of HBP ECGs could prevent adverse consequences of ECG misdiagnosis, allow more rapid global uptake of HBP, assist operators in HBP implant procedures, and facilitate management of patients with HBP devices attending centers that do not perform HBP practice. In this study, we sought to use machine learning to automate ECG analysis for HBP.

## Methods

### Case identification

**Figure 2:**
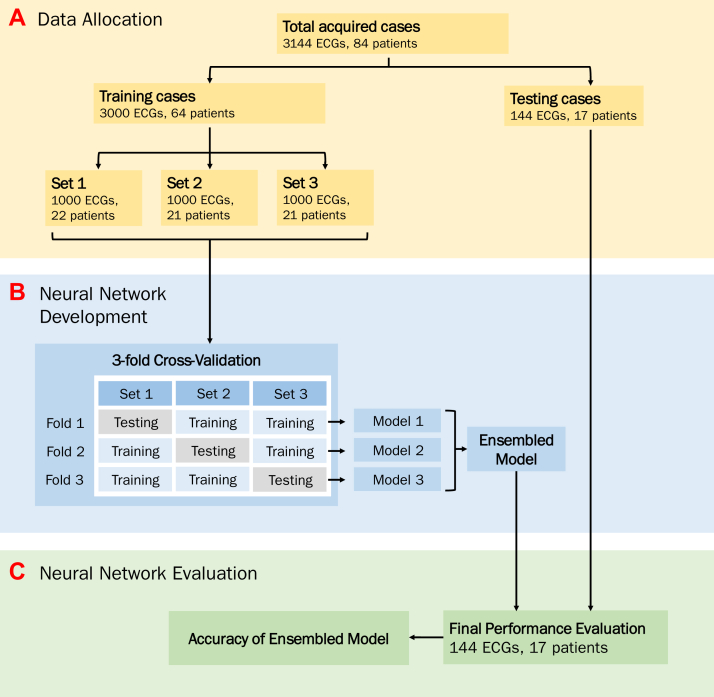
Study flowchart. ECG = electrocardiogram.

The database of electrophysiological (EP) procedures performed at Hammersmith Hospital, London, United Kingdom, was searched for HBP procedures. The research reported in this study adhered to the Helsinki Declaration as revised in 2013. Cases in which an EP system was used (Bard, Boston Scientific, Marlborough, MA) during His-bundle lead implantation were identified. Twelve-lead surface ECG recordings from the cases were inspected for paced QRS complexes, which were reviewed and classified into S-HBP, NS-HBP, or MOC. Cases were randomly assigned in a 3:1 ratio to the training or testing datasets (Figure 2). This allocation occurred at the patient level, that is, all ECGs from a given patient were assigned to the same set. The study was approved by the regional ethics board (IRAS ID: 258686).

All types of intrinsic QRS morphology were included, including narrow QRS, LBBB, and right bundle branch block (RBBB). In cases of BBB, paced QRS complexes could display any degree of resynchronization of BBB, including failed correction. All indications for HBP were included. Rare or intermediate forms of capture (eg, isolated right bundle branch capture or fusion, respectively) and left bundle branch pacing QRS complexes were excluded. Patients younger than 18 years also were excluded.

### Definitions of ECG responses

S-HBP, NS-HBP, and MOC were diagnosed by visual inspection and electronic caliper measurements of a combination of digital 12-lead surface ECGs and His-bundle lead electrograms (EGMs) during intrinsic rhythm and His-bundle lead threshold check, which included programmed stimulation when available. This was performed by a single expert in HBP ECG analysis. Criteria for diagnosing ECG responses were derived from published standardized definitions^2^^,^^13^ and are detailed in the Online Supplemental Appendix and summarized in Figure 1.

### Data extraction and segmentation

Raw, digital 12-lead surface ECG data at 1000-Hz sampling frequency were extracted from the EP system recordings for each case. Exactly 5 beats were extracted per beat class for testing set cases to ensure a balanced number of unique ECGs for the final analysis (more cases were extracted for training set cases to a maximum of 10). Each period was manually segmented using custom software^14^ into pacing artifact and paced QRS complex, the latter defined as the period from end of pacing artifact to latest QRS offset in any lead. The pacing artifact was excluded from the neural network input QRS as the size of the pacing artifact can be correlated with certain ECG responses.

### Neural network architecture and training

In this study, we used a convolutional neural network (CNN).^15^ CNNs use layers of convolutional filters to transform input data. Each layer of the CNN performs a series of “convolution” operations, which involve sliding a number of small templates, or kernels, through the output of the previous layer. Early layers work to identify the most basic features in the input data; later layers integrate these earlier findings to build complex representations of data. CNNs were inspired by the mammalian optic cortex, which Hubel and Wiesel^16^ showed contains early layers, which identify the most basic visuospatial features such as horizontal and vertical lines, and later layers, which pool these findings to identify complex shapes such as faces.

**Figure 3:**
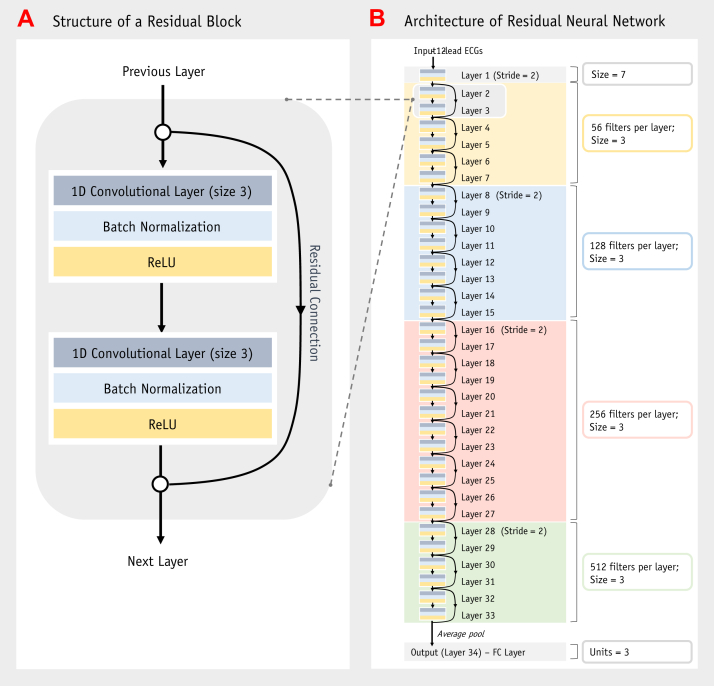
Architecture of the neural network. 1D = 1-dimensional; ECG = electrocardiogram; FC = fully connected; ReLU = rectified linear unit.

This study used a 1-dimensional CNN, in which the kernels convolve through the data in only 1 dimension, that is, they slide through the ECG over time. It was inspired by the ResNet34 architecture,^17^ a well-established 2-dimensional CNN that we modified to work with 1-dimensional ECG data (Figure 3). We trained the neural network to classify ECGs through a process termed backpropagation.^15^

We used the training data to train 3 neural networks in a process termed 3-fold cross-validation. This involved splitting the training data into 3 smaller datasets before each of the 3 neural networks was trained by using two-thirds to train the network and one-third to validate it. A different validation third was used for each of the 3 networks. The final performance of the system combined the predictions of the 3 networks (ensembling) on the unseen test set (Figure 2). All the code used to train and evaluate the networks is available online.^18^

### Visualization of learning

Examples from the testing dataset were processed to provide saliency maps.^19^ These highlighted sections of the ECG contributed most toward the final decision of the network. In brief, normally when a neural network is trained, data (such as an ECG) are fed in. Calculation occurs backward, from the output of the network, to determine how all of the parameters in the network should be adjusted so that the prediction of the neural network is more correct. However, we can extend this process of adjustment back further, beyond the first layer of the network, to the actual source data. This allows a value to be assigned to each section of the ECG that, if adjusted, would most affect the network’s decision. We use these data to highlight sections of the ECG, on the temporal axis, which are most salient. The code used to perform this process is available online.^18^

### Statistical analysis

The performance of the neural network was assessed using the Cohen kappa, which is a measure of agreement between the neural network and the true labels that adjusts for imbalance in the size of the classes. Accuracies are reported separately for each class of beats, with confidence intervals (CIs) calculated using the binomial distribution with continuity correction. Statistical analysis was performed using the scikit-learn Python package (Python Software Foundation, Wilmington, DE).

## Results

### Dataset

Seventy-six unique patients were eligible for analysis. Fifty-nine patients were randomly assigned to the training set, and the remaining 17 patients were assigned to the testing set. Baseline characteristics are given in Table 1.

**Table 1:** Baseline characteristics

	Training set (n = 59)	Testing set (n = 17)
Age (y)	72 ± 11 (40–84)	75 ± 11 (47–86)
Male	63 (83)	14 (82)
Ischemic heart disease	31 (53)	7 (41)
Ejection fraction (%)	35 ± 10 (12.5–50)	35 ± 11 (14–52.5)
Indication		
CRT	22 (37)	9 (52)
First-degree AVB	22 (37)	2 (12)
Sinus node dysfunction	11 (19)	0 (0)
High-degree AVB	4 (7)	6 (35)
Underlying rhythm		
Sinus rhythm	53 (90)	14 (82)
Atrial fibrillation	4 (7)	2 (12)
High-degree AVB	2 (3)	1 (6)
Underlying QRS morphology		
Normal	24 (34)	4 (24)
LBBB	20 (26)	8 (47)
RBBB	15 (41)	6 (35)
Cases including each ECG morphology∗		
S-HBP	37 (58)	10 (35)
NS-HBP	46 (32)	15 (54)
MOC	19 (18)	3 (11)
Total no. of ECGs†	1297	140

Values are given as mean ± SD (range) or n (%) unless otherwise indicated.

AVB = atrioventricular block; CRT = cardiac resynchronization therapy; ECG = electrocardiogram; LBBB = left bundle branch block; MOC = myocardium-only capture; NS-HBP = non-selective His-bundle pacing; RBBB = right bundle branch block; S-HBP = selective His-bundle pacing.

∗ Percentage of total number of case types.

† Cases composing the testing set were limited to contributing exactly 5 beats of each morphology to ensure accuracy measurements were balanced across patients.

### Neural network performance

**Figure 4:**
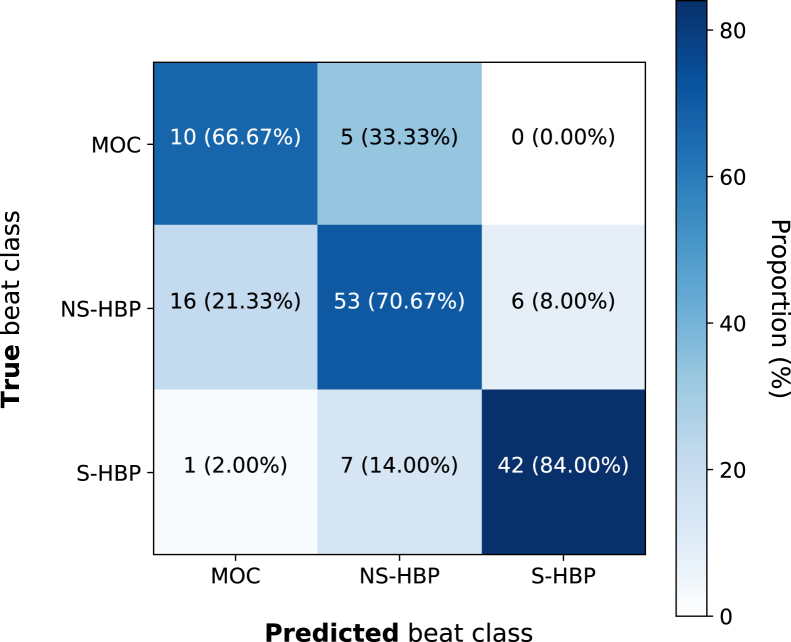
Confusion matrix for network performance. The confusion matrix shows the accuracy of the network in predicting the correct response. MOC = myocardium-only capture; NS-HBP = non-selective His-bundle pacing; S-HBP = selective His-bundle pacing.

The ensemble predictions of the 3 neural networks on the final testing set yielded a Cohen kappa of 0.59 (95% CI 0.30 to 0.88; *P* <.0001). Accuracies across the 3 classes were 84% (95% CI 70% to 92%) for S-HBP, 71% (95% CI 59% to 80%) for NS-HBP, and 67% (95% CI 39% to 87%) for MOC. Overall accuracy was 75%. Only 1 (2%) of the S-HBP ECGs was incorrectly classified as MOC, and no MOC beats were incorrectly classified as S-HBP. The confusion matrix is shown in Figure 4. Using this network’s classifications but merging S-HBP and NS-HBP into a single class produced a Cohen kappa of 0.39 and overall accuracy of 84% for discriminating MOC and HBP.

### Visualization of learning with saliency mapping

**Figure 5:**
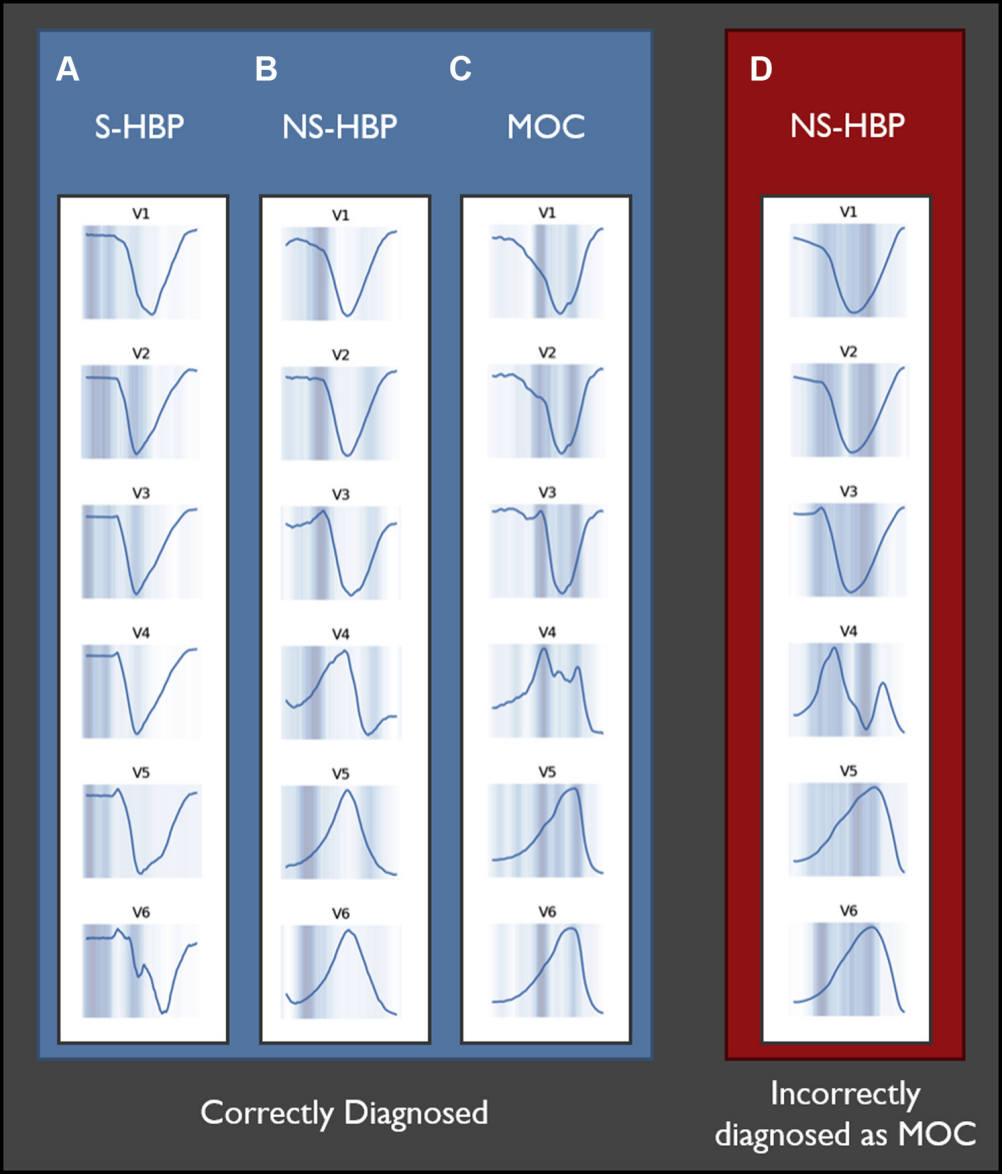
Salience maps showing neuronal activity for chest leads. *Dark blue areas* are more salient. This is an indication of which parts of the electrocardiogram (ECG) are being “assessed” by the neural network to reach a decision. **A:** Selective His-bundle pacing (S-HBP) correctly diagnosed by the neural network. The isoelectric interval appears to be salient, which is also the key feature used for human expert analysis. **B:** Non-selective His-bundle pacing (NS-HBP) correctly diagnosed by the neural network. The pseudo–delta wave appears to be salient, which is the key feature used for human expert analysis. **C:** Myocardium-only capture (MOC) correctly diagnosed by the neural network. Slurred early activation and slow late activation are salient, and both are key features for human expert analysis. **D:** NS-HBP with preservation of left bundle branch block (noncorrection) incorrectly diagnosed as MOC. This particular kind of ECG analysis also is difficult for human experts and requires analysis of threshold check transitions and intrinsic QRS morphology, neither of which is accessed by the neural network. Salience shows multiple QRS periods being assessed by the neural network, including apparent pseudo–pre-excitation and mid-QRS activity.

Examples of salience maps are shown in Figure 5. When predictions were successful, the neural network seemed to assess similar components of the ECG that are assessed by humans (Figure 1), including the isoelectric segment of S-HBP and pseudo–delta waves in NS-HBP. Incorrect predictions often showed nonspecific patterns of salience.

## Discussion

In this study, we successfully demonstrated proof of concept that a neural network can be trained to achieve automated discrimination between ECG responses to HBP. The neural network’s overall accuracy was high and significantly more accurate than chance. Although the accuracy was imperfect, with an incorrect diagnosis rate of 16% even in the most accurately diagnosed class (S-HBP), this reasonably high level of accuracy was achieved in the context of a relatively small dataset of 82 patients with intraclass heterogeneity of paced QRS morphology (varying degrees of left and right bundle recruitment). Human expert diagnosis of HBP ECGs can involve comparing the intrinsic rhythm ECG to the paced ECG, checking for transitions between capture types during threshold checks, performing EP pacing maneuvers, as well as carrying out manual measurements of key ECG intervals.^13^^,^^20^ Even when a pacemaker programming device, which is necessary for these steps, is immediately available, this process takes several minutes each time analysis is required. The neural network diagnosed capture type almost instantaneously and assessed the paced ECG alone, without access to the intrinsic and threshold ECGs. Improvement in accuracy and subsequent prospective validation are necessary for clinical use. This proof of concept justifies amalgamation of larger datasets to achieve this goal.

### HBP ECG responses

Physiological activation of the ventricles with a normal, narrow QRS in an unpaced intrinsic rhythm occurs due to rapid activation of the ventricles via the insulated His-Purkinje cardiac conduction system fibers. The variation in paced ECG responses seen with HBP are due to HBP’s fundamental property of activating the ventricles in the same way. Altering the energy output through a conventional ventricular pacing lead generally does not alter QRS morphology because only one kind of tissue can be captured: ventricular myocardium. The local myocardium activation propagates to the remainder of the ventricular myocardium nonphysiologically through slow cell-to-cell myocardial conduction. Leads positioned at the His bundle can capture 2 different tissues: conduction system tissue (the His bundle itself) and myocardium local to the lead tip. The difference in capture threshold between these 2 tissues results in the 3 potential capture types: either tissue alone (S-HBP or MOC) or both together (NS-HBP), each with different QRS characteristics (Figure 1). Correctly diagnosing which of these capture types is occurring is of crucial importance. Any misdiagnosis potentially risks programming of inappropriately high pacing output,^21^, ^22^, ^23^ which causes unnecessarily rapid battery drainage. This will result in more frequent generator replacements, each occurrence of which carries a risk of bleeding, infection, and damage to the leads. Certain misdiagnoses risk complications specific to the particular mistake.

### Distinguishing MOC from HBP

MOC does not constitute HBP and thus is rarely a desired capture type for a patient with a His-bundle pacemaker. The slow, dyssynchronous activation pattern produced is similar to conventional RV pacing.^24^ If MOC is mistakenly diagnosed as either NS-HBP or S-HBP, the patient may develop morbidity from heart failure due to pacing-induced cardiomyopathy, which also carries a mortality risk.^25^, ^26^, ^27^ HBP seems to be highly effective at preventing and treating pacing-induced cardiomyopathy, albeit in nonrandomized studies.^28^

### Distinguishing MOC from NS-HBP

Distinguishing MOC from NS-HBP usually is the hardest HBP ECG distinction for humans to make based on the paced ECG alone. Without access to threshold checks, programmed stimulation, or the intrinsic ECG, no unique feature easily discriminates the 2 capture types, unlike the isoelectric segment in S-HBP. This may offer an explanation for why NS-HBP/MOC mistakes were the most common error (60% of all errors and 23% of NS-HBP/MOC QRS complexes). MOC was also the rarest of the 3 capture types in the training set, potentially making it more difficult for the network to learn its features. This reflects the prevalence of variations in underlying anatomy. A His bundle buried deeply within the septum covered by a thick myocardium layer is a less common anatomic variant^29^ but is the variant most associated with MOC. As a result, MOC is seen less commonly than other capture types in successful HBP. In the 529-patient multicenter registry reported by Keene et al,^22^ <15% of patients exhibited MOC. Expanding the dataset may allow balancing of capture type proportions to improve this finding.

### Distinguishing MOC from S-HBP

MOC and S-HBP usually are the 2 capture types most easily distinguished from each other by human analysis, particularly when the intrinsic QRS is narrow. Due to the absence of myocardial capture, S-HBP produces a characteristic isoelectric segment before QRS onset that is easier to identify than the subtle morphologic changes or cumbersome measurements that distinguish MOC from NS-HBP. In combination with this difference, the QRS of S-HBP is more clearly different from MOC than it is from NS-HBP due to the considerable difference in ventricular activation pattern. Furthermore, due to the nature of myocardial and conduction system thresholds (detailed in the Online Supplemental Appendix), both MOC and S-HBP cannot be seen in the same pacing configuration in a particular position at any given time, giving humans with this understanding an advantage when analyzing a threshold check from a lead. Even without this advantage, S-HBP was the most successfully diagnosed capture type by the neural network, and only 1 (1.5%) QRS complex of the group of MOC and S-HBP QRS complexes in the testing set was incorrectly diagnosed as the other.

### Distinguishing S-HBP from NS-HBP

Although a randomized controlled trial is awaited, there is a physiological basis and nonrandomized evidence for the superiority of S-HBP and NS-HBP over MOC and other sites of RV pacing. However, the differences between S-HBP and NS-HBP are an area of ongoing scientific enquiry.^24^^,^^30^^,^^31^ NS-HBP allows continued pacing if infrahisian conduction block occurs, whereas this could be symptomatic or even fatal with S-HBP. Therefore, distinguishing between S-HBP and NS-HBP ECG appearances is clinically important for programming in patients at risk for infrahisian conduction block. The putative safety of nonselectivity in this scenario is due to simultaneous capture of myocardium. Whether the fact that some myocardium is activated through cell-to-cell myocardial conduction means that dyssynchrony occurs, risking pacing-induced cardiomyopathy, has been debated.^24^

Electrical and mechanical synchrony assessment of the left ventricle suggests that NS-HBP does not induce left ventricular dyssynchrony, but RV synchrony likely is affected.^6^^,^^24^^,^^31^ The importance of this finding is not completely clear, but a nonrandomized comparison between S-HBP and NS-HBP found no statistically significant difference in outcomes between them.^30^ The current consensus among the HBP community is that NS-HBP does not risk adverse consequences unless the intrinsic His-ventricular (HV) interval is very long or the rare circumstance in which patients are highly sensitive to minimal dyssynchrony. True delta waves from an accessory pathway have been reported to induce ventricular dysfunction in rare cases.^13^^,^^21^ Such circumstances further support the importance of discriminating between S-HBP and NS-HBP.

The neural network mistook selective and non-selective HBP for each other at a relatively low rate: 10% of the group of S-HBP and NS-HBP beats in the testing set. These errors occurred at a higher rate than MOC/S-HBP mistakes and at a lower rate than MOC/NS-HBP mistakes, both as a proportion of all errors and as a proportion of grouped class sizes. This reflects the intermediate difficulty of this comparison for human analysis. Differentiating S-HBP from NS-HBP relies on identifying an isoelectric segment or pre-excitation, respectively, between the pacing artifact and QRS onset. This can be very clear due to considerable pre-excitation (eg, due to high pacing output with long HV interval) or, conversely, a definitively flat, physiological isoelectric stimulus-V segment in all leads. However, pre-excitation can be almost imperceptibly subtle, and a wandering baseline can mimic pre-excitation.

### Why CNNs may excel at ECG analysis

CNNs have been used successfully in the past to classify biological waveform data such as ECGs,^32^ encephalograms,^33^ aortic pressure waveforms,^8^ and even sleep sounds.^34^ CNNs are not the only approaches that can be used for waveform classification, but they have rapidly become the state of the art for analyzing 1-, 2-, and 3-dimensional medical data. Their structure is inspired by the mammalian optic cortex, in which a series of layers work to extract increasingly complex visuospatial structures from input data. Indeed, the only way the neural network is able to perform its task is by learning patterns of small templates, termed kernels, which it can slide through data to identify matching areas of interest. This introduces limitations on how the CNN is able to process data, as each kernel is forced to extract data only from within its own small “visual field.” These limitations actually strengthen the performance of these networks because they hamper the ability of the neural network to merely memorize, or “overfit,” examples it has seen. For example, a CNN is not capable of simply comparing the precise voltage at 2 specific time points to recognize a specific ECG, unlike more traditional neural networks. This allows CNNs to be highly efficient and to learn to extract data in a way that generalizes to unseen examples. Interestingly, we found that neural networks with >34 layers performed worse on our testing set, presumably as the ability of “deeper” networks to overfit to ECGs dominated any improvements in the processing capabilities.

### Understanding neural network predictions

It is intuitive to explain the network’s variable predictive ability as mimicking human performance. First, the neural network’s learning process is thought to be analogous to that of the human brain. Second, humans find certain classifications easier than others due to inherent features and defining criteria of those classes (eg, MOC vs S-HBP). However, there are other potential reasons for the network’s performance being in line with expected human performance. Class imbalance might hamper the network’s ability to learn less common classes and may bias the network toward predicting more common classes. When humans find some classification tasks difficult and the labels for network training and testing are based on human assessment, labeling errors will occur at a higher rate in the classification tasks that are more difficult for humans (eg, NS-HBP vs MOC in patients with failed correction of LBBB). This will worsen network performance for those classifications.

The nature of neural network machine learning has been characterized as a “black box.”^35^^,^^36^ This refers to the apparent impossibility of scrutinizing how a neural network makes predictions. Human predictions often rely on overt criteria or judgments that can be expressed, but they also can rely on pattern recognition or other heuristics that are not easily scrutinized. Salience mapping offers a window into some aspects of neural network predictive processes. Examples of successful prediction show that the neural network seems to assess similar aspects of ECGs that are the focus of human assessment (Figure 5).

### Potential role of automated HBP ECG analysis

A neural network trained to very high accuracy has several potential applications and benefits. The HBP procedure can be performed in catheterization laboratories with EP systems, where His-bundle lead EGMs are visualized alongside 12-lead ECGs, and electronic caliper measurements can be made across EGMs and ECGs, including the important H-QRS_end_ time. However, most HBP implantations are performed in catheterization laboratories that are not dedicated to EP procedures, so the 12-lead ECG is not visualized alongside the EGM. Making measurements in this scenario can be cumbersome. Automated analysis would greatly facilitate intraprocedural diagnosis in such laboratories, potentially shortening procedural time. HBP follow-up also requires accurate diagnosis of ECGs, which currently restricts practice to centers with considerable HBP expertise. Automated diagnosis could help to democratize HBP to be practiced at any center. Finally, even experts occasionally make errors that lead to inappropriate programming, with potentially detrimental clinical consequences. Neural networks are not prone to errors that humans might make due to tiredness, distraction, or lack of concentration, minimizing errors overall.

### Study limitations

ECG diagnosis for label determination was performed by a single expert. Although objective HBP criteria have been set out in detail and are recognized internationally as the gold standard, cases remain for which diagnosis is uncertain even when experts adhere strictly to these criteria. For example, NS-HBP with partial correction of LBBB can be challenging to distinguish from MOC. Both exhibit LBBB-like morphology, and their ECG capture measurement ranges overlap (for full definitions see the Online Supplemental Appendix). Transitions in morphology during threshold checks can be due to variable bundle recruitment rather than loss of His-bundle capture. Single raters are likely to make some errors even if expertise minimizes this possibility, and there can be inter- and intra-rater reproducibility issues with ratings that are masked by single assessments by single raters. Future work will involve ratings by multiple experts to establish consensus in uncertain cases and eliminate labeling errors. The next phase of development also will involve training the network to recognize ECG responses not assessed in this study, such as noncapture, native conduction, and fusion.

There was considerable class imbalance between the types of capture (S-HBP, NS-HBP, MOC) within the training and testing datasets. Although the Cohen kappa performance metric accounts for such imbalances when reporting results, these disparities nevertheless will compromise the neural network’s ability to learn the rarer classes of data.^37^ Indeed, MOC cases composed the smallest category of data, and the neural network performed more poorly in identifying these beats (67% for MOC vs 84% for S-HBP). Future endeavors will focus on broadening the size of the datasets and addressing these class imbalances. Intrinsically narrow QRS, LBBB, and RBBB all were included in the dataset, and partial, full, or no correction of BBB all were included. Although this introduced intraclass heterogeneity, it allows more universal application of the algorithm. Future work will allow diagnosis of BBB correction. No particular subgroup demonstrated particularly poor or high accuracy in the testing set.

This iteration of the machine learning algorithm required manual QRS segmentation, but this process can be automated through conventional computational means or machine learning. Raw digital ECG data were the data source in this study, but images of ECGs could be automatically transformed into equivalent signals to be input into the network. A data pipeline for this is under development.

This was a single-center retrospective study design with a relatively small dataset, and the cases included were biased toward certain indications due to ongoing research into HBP during the study period. The most common indication for HBP in international multicenter registries is high-degree atrioventricular block, but this was the least common indication in our study. This was mitigated by the balanced proportions of intrinsic RBBB, LBBB, and narrow QRS within and between the training and test sets. This study demonstrated proof of concept justifying dataset expansion. Indeed, at present the threshold of accuracy (or kappa) such a classifier must obtain to be clinically superior to an expert human is unknown, as no data exist demonstrating the performance of humans at this task outside of the research environment. A highly accurate, fully automated, machine learning algorithm trained on large-scale, multiple rater–labeled data will be tested in a prospective, multicenter validation study on consecutive cases and compared to human expert analysis.

## Conclusion

We demonstrated proof of concept that discrimination between HBP ECG responses can be automated using machine learning. When a larger dataset is used to train the network to higher accuracy, automated AI ECG analysis could facilitate HBP implantation and follow-up and prevent complications from HBP ECG misdiagnosis.

## Funding Sources

Dr Arnold (P74166) acknowledges support from the National Institute of Health Research (NIHR) Imperial Biomedical Research Centre (BRC) and the British Heart Foundation (BHF) Imperial Centre of Research Excellence (RE/18/4/34215). Dr Howard is supported by the Wellcome Trust (212183/Z/18/Z). Prof Francis (FS 04/079) and Dr Whinnett (FS/13/44/30291) have received individual funding from the BHF.

## Disclosures

The authors have no conflicts of interest to disclose.
