# Corrigendum to: Discriminating electrocardiographic responses to His-bundle pacing using machine learning [Cardiovascular Digital Health Journal 1 (2020) 11–20/5]

**DOI:** 10.1016/j.cvdhj.2020.10.002

**Published:** 2020-10-14

Ahran D. Arnold, MBBS, James P. Howard, MB BChir, Aiswarya Gopi, BSc, Cheng Pou Chan, BSc, Nadine Ali, BMBCh, Daniel Keene, MBChB, PhD, Matthew J. Shun-Shin, BMBCh, PhD, Yousif Ahmad, BMBS, PhD, Ian J. Wright, BSc, Fu Siong Ng, MBBS, PhD, Nick W.F. Linton, MBBS, PhD, Prapa Kanagaratnam, MB BChir, PhD, Nicholas S. Peters, MBBS, MD, FHRS, Daniel Rueckert, PhD, Darrel P. Francis, MB BChir, MD, Zachary I. Whinnett, BMBS, PhD

From the National Heart and Lung Institute, Imperial College London, Hammersmith Hospital, London, United Kingdom.

DOI 10.1016/j.cvdhj.2020.07.001

This article was initially posted with the third author incorrectly listed as Aiswarya A Gopi. The author name has since been corrected online as Aiswarya Gopi so please use this corrected version for citation purposes.

