# Supplementary material for: Discriminating electrocardiographic responses to His-bundle pacing using machine learning
Source: Cardiovasc Digit Health J. 2020 Aug 26;1(1):11–20. doi: 10.1016/j.cvdhj.2020.07.001 (PMC7484933; doi:10.1016/j.cvdhj.2020.07.001)
Supplement: Online Supplemental Appendix [file mmc1.docx]

Supplementary Appendix

# Neural Network Training

We trained the neural network by feeding in ECG data in batches of 16 ECGs, with traces zero-padded to be equal length within a batch. ECGs were normalized lead-wise according to the median and interquartile range of voltages across the training dataset. We calculated loss over each batch using the categorical cross entropy loss function with label smoothing (ɑ=0.1) and online hard example mining (rate=0.9). Weights were updated with the AdamW optimizer with a learning rate of 0.001 and weight decay of 0.01. The CNNs were created using the Python programming language (Python Software Foundation, Delware, United States) and the Pytorch machine learning framework, and were trained using an RTX Titan graphical processing unit (NVidia Corporation, California, United States).

# Definitions of ECG Responses

• **Selective HBP** (S-HBP) occurs when the His bundle is captured alone without any local myocardial capture. Both ventricles are activated entirely by the conduction system. The paced QRS is similar to intrinsic QRS in patients with intrinsically normal QRS complexes, except for the presence of a pacing stimulus artefact followed by an isoelectric segment that precedes the QRS complex. In bundle branch block (BBB) the appearance of the QRS is determined by the degree of BBB correction.

• **Non-Selective HBP** (NS-HBP) occurs when both the His bundle and local myocardium adjacent to the lead tip are captured. The paced QRS shows a slurred onset, beginning very soon or immediately after the pacing artefact (in contrast to the iso-electric segment seen in S-HBP). This is caused by slow cell-to-cell myocardial propagation in the region of myocardium close to the lead tip (the basal RV septum). The remainder of the QRS can be similar to the intrinsically normal QRS, as the conduction system activated the rest of the ventricles rapidly. However, in BBB correction, the entire QRS appearance can be very different to intrinsic QRS.

• **Myocardium Only Capture** (MOC) occurs if the His bundle is not captured and only the local myocardium is captured; this is also termed “myocardial capture,” or “septal capture”. Similarly to NS-HBP, the paced QRS of MOC begins very soon or immediately after the pacing artefact, however the QRS produced is usually broad as the entire ventricular myocardium is activated by slow cell-to-cell myocardial propagation. The activation pattern is similar to right ventricular septal pacing. This can be difficult to distinguish from NS-HBP, particularly when LBBB is partially corrected or uncorrected. MOC does not constitute HBP but is seen during attempted HBP and leads that produce NS-HBP may produce MOC at lower outputs.

The threshold refers to the minimum energy requirement to sufficiently depolarise the tissue such that it is activated, resulting in spread of activation in that tissue. The combination of capture types observed when pacing in a particular position with a given pacing configuration depends on the relative threshold energies of the two tissues.

When the myocardial threshold exceeds the His bundle threshold, S-HBP can be observed at pacing outputs above the His bundle threshold but below the myocardial threshold. At higher pacing outputs (above both thresholds), NS-HBP is observed. Conversely, when the His bundle threshold exceeds the myocardial threshold, MOC can be observed at pacing outputs above the myocardial threshold but below the His bundle threshold. At higher pacing outputs, NS-HBP is observed. Thus MOC and S-HBP cannot both be seen in the same pacing configuration in a particular position at any given time.

Definitions of ECG response vary depending on whether the intrinsic QRS is normal or if bundle branch block (BBB) is present. The following definitions are derived from internationally recognised, standardised definitions. EGM criteria have also been set out and were used for diagnosis as well as programmed stimulation where available.

## Interval Definitions

**His-Ventricular Interval (HV):** duration from the onset of the His signal on the His bundle lead electrocardiogram (EGM) to the earliest onset of QRS complex in any lead of 12-lead ECG.

**Stimulus-to-Ventricular-Onset Interval (Stim-V):** duration from onset of pacing artefact to the earliest onset of QRS complex in any lead of 12-lead surface ECG.

**His-to-QRS-offset (H-QRS_end_):** duration from onset of His signal on the His bundle lead EGM to the latest offset of QRS complex in any lead of 12-lead surface ECG.

**Stimulus-to-QRS-offset (Stim-QRS_end_):** duration from onset of pacing artefact to the latest offset of QRS complex in any lead of 12-lead surface ECG.

**QRS duration (QRSd):** duration from earliest onset of QRS in any lead of the 12-lead surface ECG to latest QRS offset in any lead of the 12-lead surface ECG.

**QRS morphology (QRSm):** Pattern of QRS appearance e.g. pre-excited, left bundle branch block (LBBB), right bundle branch block (RBBB).

## ECG responses for narrow intrinsic QRS

For **S-HBP** to be diagnosed all of the following criteria had to be met: 1) Stim-QRS_end_ = H-QRS_end_, 2) Stim-V = HV, 3) Intrinsic QRSd = paced QRSd, 4) Paced QRSm identical to intrinsic QRSm, 5) S-HBP threshold must be the lowest threshold if two thresholds are observed.

For **NS-HBP** to be diagnosed all of the following criteria had to be met: 1) Stim-QRS_end_ = H-QRS_end_, 2) Stim-V < HV and < 40ms, 3) Intrinsic QRSd < paced QRSd by ≤ HV, 4) Intrinsic QRSd paced QRSd, 5) Paced QRSm appearance of pre-excited intrinsic QRSm, 5) NS-HBP threshold must not be the lowest threshold if two thresholds are observed.

For **MOC** to be diagnosed all of the following criteria had to be met: 1) Stim-QRS_end_ > H-QRS_end_, 2) Stim-V < HV and < 40ms, 3) Intrinsic QRSd < paced QRSd, 4) Paced QRSm appearance of LBBB-like morphology, 5) MOC threshold must be the lowest threshold if two thresholds are observed.

## ECG responses for intrinsic LBBB

LBBB introduced more complexity to ECG diagnosis for two reasons. Firstly, the possibility of correcting LBBB, either partially or fully, by HBP results in variable responses from both S-HBP and NS-HBP. Secondly, MOC and NS-HBP (partially when partial correction occurs and/or when MOC produces a more synchronous activation than intrinsic LBBB) potentially overlap in many characteristics including Stim-V, Stim-QRS_end_, QRSd and QRSm. Therefore care must be taken to differentiate the two.

For **S-HBP** to be diagnosed all of the following criteria had to be met: 1) Stim-QRS_end_ ≤ H-QRS_end_, 2) Stim-V ≤ HV, 3) Intrinsic QRSd ≥ paced QRSd, 4) Paced QRSm either identical to intrinsic QRSm, if no correction of LBBB has occurred, or approaching narrow QRS if correction has occurred.

For **NS-HBP** to be diagnosed all of the following criteria had to be met: 1) Stim-QRS_end_ ≤ H-QRS_end_, 2) Stim-V < HV and < 40ms, 5) Paced QRSm appearance of pre-excited LBBB if no correction of LBBB has occurred, or approaching pre-excited narrow QRS if correction has occurred, 5) Transition between NS-HBP and MOC on threshold check or programmed stimulation.

For **NS-HBP** to be diagnosed all of the following criteria had to be met: 1) Stim-V < HV and < 40ms, 2) Paced QRSm appearance of LBBB-like morphology, 5) Transition between NS-HBP and MOC on threshold check or programmed stimulation.

## ECG responses for intrinsic RBBB

RBBB introduces more complexity to ECG analysis due to the possibility of correcting RBBB. S-HBP may result in full, partial or no correction. NS-HBP is always expected to produce some degree of RBBB resynchronization. MOC is usually easier to differentiate from NS-HBP due to the change to LBBB-type morphology.

For **S-HBP** to be diagnosed all of the following criteria had to be met: 1) Stim-QRS_end_ ≤ H-QRS_end_, 2) Stim-V ≤ HV, 3) Intrinsic QRSd ≤ paced QRSd, 4) Paced QRSm identical to intrinsic RBBB if no correction of RBBB has occurred or approaching narrow QRS if correction has occurred.

For **NS-HBP** to be diagnosed all of the following criteria had to be met: 1) Stim-QRS_end_ < H-QRS_end_, 2) Stim-V < HV and < 40ms, 3) Paced QRSm appearance approaching pre-excited narrow QRS

For **MOC** to be diagnosed all of the following criteria had to be met: 1) Stim-QRS_end_ > H-QRS_end_, 2) Stim-V < HV and < 40ms, 3) Paced QRSm appearance of LBBB-like morphology
